# Impacts of COVID-19 Pandemic on Psychological Well-Being of Older Chronic Kidney Disease Patients

**DOI:** 10.3389/fmed.2021.666973

**Published:** 2021-05-26

**Authors:** Alex Siu Wing Chan, Jacqueline Mei Chi Ho, Jane Siu Fan Li, Hon Lon Tam, Patrick Ming Kuen Tang

**Affiliations:** ^1^Department of Applied Social Sciences, The Hong Kong Polytechnic University, Kowloon, Hong Kong; ^2^School of Nursing, The Hong Kong Polytechnic University, Kowloon, Hong Kong; ^3^Department of Anatomical and Cellular Pathology, State Key Laboratory of Translational Oncology, The Chinese University of Hong Kong, Hong Kong, China; ^4^Education Department, Kiang Wu Nursing College of Macau, Macao, China

**Keywords:** COVID-19 pandemic, psychological well-being, aging-old age-seniors, immune system, chronic kidney disease

## Abstract

COVID-19 pandemic has been a major global issue, its eventual influences on the population welfare, global markets, public security, and everyday activities remain uncertain. Indeed, the pandemic has arisen a significant global threat. Its psychological impact is predicted to be severe and enduring, but the absolute magnitude is still largely unclear. Chronic kidney disease (CKD) is a complication markedly contributes to the mortality of COVID-19 cases, meanwhile several studies have demonstrated the high frequency and seriousness of the COVID-19 in CKD patients receiving dialysis. Importantly, the influence of COVID-19 among CKD patients without dialysis is still largely unexplored. Thus, we systemically summarized how mental health affects the spreading of COVID-19 to virtually worldwide, covering perspectives from several countries across a wide range of fields and clinical contexts. This review aims to provide the latest details and reveal potential concerns on the public health including psychological well-being of the older patients with CKD.

## Introduction

Psychological well-being (PWB) is fundamentally equivalent to other phrases that apply to desirable psychological operations, including pleasure or fulfillment. It is not essential or valuable to consider the fundamental differences between all these phrases (1). Psychological well-being means being on good terms with others and leading a purposeful and meaningful life (2). It was found that people with positive psychological well-being are more carefree and enjoy a more vibrant and comfortable life (3). However, nearly 25% of people with chronic conditions experienced psychological problems related to COVID-19, particularly CKD patients (4, 5). Currently, personalized treatment should be the norm in handling CKD patients (6–10). Because of COVID-19, it seems to be far more critical that this approach be pursued to minimize the possibility of excessive or insufficient treatment and reduce the likelihood of developing a prejudice (11). This applies to COVID-19 as people's psychological well-being experienced the most significant impact during the pandemic. The ones with stable psychological well-being were in a better state than those whose well-being was below par (3). As there have been constant interruptions to everyday life owing to social distancing, which has been imposed to minimize the transmission of COVID-19, precedent hazards to public mental health were observed (12). The risk of COVID-19 severe complications and poor prognosis is higher for CKD patients, particularly those who undergo chronic dialysis therapy, including higher rates of hospitalization, intensive-care unit admission, mechanical ventilation, and death (13). The well-being of patients has been a significant issue during the pandemic considering the mental effects on even ordinary healthy people were more critical than expected (14). Hence, the impact on the psychological well-being of older CKD patients will be studied.

## Interaction Between COVID-19 AND Psychological Well-Being

The findings of the research conducted by Moreno et al. (15) are diverse (Table 1), possibly due to variations in the methodology adopted, the venues of the analysis, and the fact that the research takes place during the pandemic. Possible consequences of modifications to health resources on accessibility and reliability and performance of psychiatric services throughout the COVID-19 pandemic (16). Phobic anxiety, impulse purchase, and television addiction, all linked to psychological disruptions, insomnia, exhaustion, and consciousness deterioration, have been documented, and digital networking has been linked to heightened anxiety and depression-associated anxiety (17–19).

**Table 1:** Implications for medical resources changes on availability, efficiency, and output of psychological treatment during the COVID-19 pandemic.

	**Possible negative consequences**	**Possible positive consequences**
The healthcare system's primary emphasis on the detection, reduction, and control of COVID-19.	• Primary educational emphasis on physical wellbeing; emphasis on social distancing rather than bodily distancing while remaining linked. • Redistribution of services to meet physical wellbeing treatment demands; reduced face-to-face interactions among and inside care units; bodily and psychological toll on medical staff; personnel deficiencies in medical services.	• Knowledge about the psychological implications of COVID-19 has the potential to raise the public's general psychological health awareness; the chance to highlight the significance of self-care, recovery measures, and household assistance; enhancement of funding for psychological health care from non-profit or private institutions; and multidisciplinary initiatives to activate support groups, using innovations to enable swift, scalable, and effective team interaction and collaboration inside and among teams (for example, psychological well-being, and basic treatment). • Improve bodily and psychological well-being through behavioral changes, the implementation of low-barrier destigmatized psychosocial assessments, counseling programs, and a student-to-student framework.
Controlled admission to other kinds of medical services as a critical component of COVID-19 management	Triage procedures that prioritize acute patients only resulted in a reduction in hospital visits (such as those for administering or distributing of drugs), emergency department visits, inpatient treatment, and pharmaceutical accessibility; community psychoeducation, community cognitive treatment, and mutual help initiatives being eliminated or scaled down; options for cardiometabolic and detrimental impact screening being reduced, overall inpatient spaces being reduced; hospital entry restrictions; reduced hospitalizations; hasty departure to mitigate the possibility of healthcare facility-related infection, particularly for those who have been hospitalized involuntarily.	Re-evaluation of the effective provision distribution, data retention regulations, and payment for telehealth and multimedia, virtual medical services, and choices for in-home care; availability strategies (for example, web channels), health policy, privacy rules, flexible drug coverage, including the usage of restricted drugs; creation of digital platforms for community outpatient therapies; controlling techniques that are less resistant to risk; less overcrowding in inpatient wards; re-evaluation of the duration of inpatient stays that are required; reassessment of the demand of forced medical services

*Source: Moreno et al. (15)*.

The illustration (Table 1) shows the possible effects of modifications to health resources on psychiatric services throughout the COVID-19 pandemic. It further describes the reliability and impact of these adjustments in resources amid the COVID-19 pandemic (13). Numerous people worldwide are now feeling stress and paranoia, particularly the elderly or people with existing health issues and even active and energetic youths. The anxiety is about the novel coronavirus, which the technical term is severe acute respiratory syndrome coronavirus (SARS-CoV-2) (20). This hideous virus induces a lethal respiratory condition known COVID-19, which brings fever, severe chest infections, and breathlessness (occasional lack of taste and smell or digestive troubles). COVID-19 is a disease that can escalate quickly; in certain instances, it can be fatal (21). Therefore, the psychological well-being of CKD patients during the period of COVID-19 should be concerned. The current situation is difficult for everyone in public, particularly for the older people who are existing mental health issues; such as anxiety and depression-associated anxiety that are more vulnerable to major medical problems related to coronavirus infection and the emergence of COVID-19 pulmonary disease with possibly catastrophic results (22).

## Effects of Lockdown on Humans Amid COVID-19

Lockdown may also lead to pressure, resentment, and intensified harmful activities like internet gambling. In earlier outbreaks, the older person affected by lockdown had a higher likelihood of experiencing psychological problems and sorrow (23, 24). It has been observed that the number of elderly resorting to counseling services because of psychological distress has increased (25, 26). Local personal networks and experiences with other inhabitants, relatives, and caregivers due to isolation could also contribute to depression, immobility, and an inactive lifestyle among citizens, adding to their solitude. Solitude as well as social alienation have been associated with worse psychological health (for example, stress, despair, and neurological damage) along with the reduced quality of life (for example, weaker motor control, poorer heart health, sleep disturbance, and loss of strength) and increased death rates. Forced alienation may very well contribute to an inactive lifestyle, yet a person's lifestyle is crucial to reducing physical, mental, and societal medical issues (27, 28).

Based on current evidence from past disease outbreaks and new data from the recent episode, it is anticipated that mental morbidity will eventually increase. Also, such morbidity may escalate afterward and last longer than the external harmful effects of the outbreak (29). Such a pattern is shown in various aspects throughout this edition, which states that the initial stages of the epidemic did not automatically trigger a rise in psychological well-being sessions. Nevertheless, transition to the current constraints introduced by COVID-19 has added burdens to the field of psychological well-being (30). Moreover, the predicted rise in psychiatric illness, which could lead to more suicidal behavior, is more likely to emerge during and after the outbreak, as the financial crisis, local mental health services, human weaknesses, and the harsh truth of radically transformed habits converge (31).

## Health Effects of COVID-19: Worldwide Situation

The World Health Organization states that the global transmission of COVID-19 is accelerated bit by bit. As shown by the data updated on 18 February 2020, the total number of confirmed instances had reached over 72,000, with almost 1,900 coming from China (32). The total number of deaths from COVID-19 is estimated to be more significant since the estimates vary from country to country. Although the virus affects everyone, assuming that all factors are identical, evidence has consistently shown that the death rate is higher among older individuals and people with complications (3, 33). The case fatality rate (CFR) of individuals aged 70 was between 0.3 and 3.5%. These figures are lower than the 8% CFR in patients between 70 and 79 and ~15% in patients over 80 in China. As for Italy, empirical studies indicate the average age of patients dying from COVID-19 was 80, with CFR rising above 70 years of age; 12.5% (34–43), 19.7% (44–53), and 22.7% (over 90) (54). A study found that found the subjects to have obtained COVID-19-related information once in a while from the following channels: online (including sites, online news, and internet networks, such as Facebook and Twitter), acquaintances, traditional media (including television, newspapers, and radio), structured activities on COVID-19 (be it online or face-to-face), medical workers in healthcare environments, colleagues, and families. This research concluded that about 80% of the subjects received COVID-19 information online (55).

Psychological symptoms and illnesses can occur as an adjunct to an unavoidable disease incident. These can appear at an active stage or later in time. The outbreak itself is a traumatic event, but it is significantly more disturbing to work as a medical practitioner to cope with such a severe disease (56). In America, the fatality rate in New York City for patients 75 years old or older was over 1,500 per 100,000 people. Advanced age and complications such as coronary disease, diabetes, chronic lung ailments, and persistent kidney dysfunction tend to increase the dangers of COVID-19. In many more existing patients with renal failure, where the health care system may evaluate employees, The CKD patients should consider the risks of death from COVID-19 in assessing the risks and benefits of treatment options (57).

## Analyzing the Psychological Issues Caused by COVID-19

Controversial expectations, coping with significant shortcomings in the resources for screening and therapy and the protection of patients and health care professionals from infection, the pressure of current overall well-being measures that place restrictions on personal autonomy, tremendous and rising financial troubles, and contradictory instructions from professionals are some of the major factors which would inevitably contribute to endless difficulty and heightened threats of Covid-19-related psychological disorder (58, 59). Healthcare practitioners have a major role in resolving these serious consequences as part of the pandemic response (60, 61).

Wide analysis of poor mental health has proven that tremendous problems are widespread in infected communities, an observation that is likely to be replicated in populations affected by the Covid-19 pandemic. Owing to these challenges, numerous people refuse to give in to therapy. Some individuals have developed new attributes. Considering all factors, in “standard” disastrous incidents, technological failures, and deliberate events of massive destruction, a major concern is post-traumatic stress disorder (PTSD) arising from exposure to trauma. Contagious illnesses, such as dangerous virus infection, may not follow existing trauma frameworks required to study PTSD, but other psychological issues such as stress and anxiety can arise (62). Several communities could be more vulnerable than others in coping with the psychosocial effects of disease outbreaks. Particularly, people who suffer from the disease, those at increased risk for infection (including the elderly, people with lower resistance, and those residing in community surroundings), and people with prior medical, emotional, or drug use problems are at heightened risk for antagonistic psychosocial outcomes (63).

## Older Ckd Patients During COVID-19 Pandemic

The COVID-19 pandemic revealed the notorious vulnerability of the monetary system and the overwhelming ramifications for our financial structure should new tactics not be adopted to tailor medical services to specific patient subgroups. The massive group of elderly and feeble people over 65 poses a public health concern. According to the latest data from the Istituto Superiore di Sanità of Italy, COVID-19 tends to be more deadly among elderly patients: 96.4% of deaths were over 60 years of age. People aged 70 or above account for 35.5% of instances as statistics were classified by age level, whereas participants aged over 80 accounts for 52.3%. Patients with renal disease are an elderly group that is especially susceptible to infection and carries a higher risk of death than the average person. The massive group of elderly and feeble people over 65 poses a public health concern. The COVID-19 pandemic revealed the notorious vulnerability of the monetary system and the overwhelming ramifications for our financial structure should new tactics not be adopted to tailor medical services to specific patient subgroups (64).

Given that most patients with CKD are seniors, who experience biological deterioration of renal function and are more vulnerable to renal disease, COVID-19 emerges as a pertinent issue because of the heightened risk of comorbidities and fatality in patients with chronic renal disease (65). Moreover, specific antiviral and immunosuppressive approaches to combat COVID-19 infection have been hampered by severe renal damage. The combination of age and chronic renal disease is most likely a possible cause of COVID-19 in immunosuppressive activity. Immunol senescence is a condition that occurs in older adults and is accompanied by weakened responsive and inherent immune function (66). Numerous changes have occurred, including thymic involution, a reduction in naïve T-cells and progenitor B-cells, and a reduction in the production of MHC class II on macrophages. Among chronic renal disease cases, a significant immunosuppressive condition has been observed as well: (i) diminished granulocyte and monocyte/macrophage phagocytic activity; (ii) reduced antigen-presenting potential of antigen-presenting cells; (iii) loss of antigen-presenting dendritic cells; (iv) weakened B lymphocyte numbers and immune generating ability; (v) reduction in naïve and central memory CD4^+^ and CD8^+^ T lymphocytes; (vi) disrupted cell-mediated resistance. Because of such considerations, older CKD patients must fully adhere to the guidelines of the Ministry of Health and Nephrological Scientific Societies for COVID-19 reduction(34–53, 67–92).

## Challenges And Recommendations Concerning the Psychological Impact Among Ckd Patients

Under the outbreak, further attention to be paid to public health, both physical and psychological, to help communities during this challenging period (71–73). The COVID-19 outbreak has brought many extra challenges to the study, planning, and management of health (4, 74, 75). The problems of COVID-19 mental health and bureaucratic responses to the outbreak are not exactly unprecedented. Past mental health deficiencies could become more deep-rooted and considerably more difficult to tackle (76, 77). Evidence from all over the world of shifts in individuals' mental health, possibly attributable to the COVID-19 outbreak, has been hindered by the use of residence assessments, distorted or unverifiable mental health metrics, and the lack of other pre-COVID-19 conventional knowledge to measure the transition, be it among individuals or throughout the whole population (78, 79, 93–95).

One study showed elevated rates of mental illness among US adults in 2020 compared with 2018, and the increase was the most significant among young adults aged between 18 and 24 and females (19). Legislators, politicians, and specialized agencies require accurate information on the shifts in mental health associated with the outbreak so that decisions are backed by knowledge on the extent of transitions in individuals' mental health and vulnerability to psychiatric problems (80). In such a crisis, the ability to track and address psychosocial needs during proper consultation in clinical care is severely constrained by the immense complexity of household regulation. Strategies for telemedicine are given to psychosocial institutions and are increasingly distributed in stimulating environments (81). As far as COVID-19 is concerned, psychosocial evaluation and monitoring may include concerns linked to COVID-19 stress factors (e.g., exposures to infected materials, infected family members, loss of loved ones, and segregation) and further mishaps (e.g., economic hardship) (82, 83).

Psychosocial impacts include depression, stress, psychiatric disturbances, insomnia, heightened drug use and aggressive actions at home, and signs of vulnerability (84) (Table 2), such as previous physical or emotional disorders. Some individuals may need guidance regarding structured psychological health evaluation and treatment. Others benefit from ongoing counseling to enhance health and facilitate adjustment (e.g., psychoeducation or cognitive behavior approaches) (85, 86). Given the increasing financial crisis and the multiple threats of this outbreak, self-destructive thoughts may emerge, which entails a timely meeting with professionals or a recommendation for possible crisis psychiatric hospitalization (87, 88). At the gentler end of the psychosocial spectrum, a large amount of interaction between patients, families, and the wider populace could be better structured by presenting evidence on typical reactions to this form of resistance and by drawing attention to what people can do in the middle of severe circumstances (89, 90).

**Table 2:** Description of studies on older CKD patients negative psychological impact during COVID-19 in the review.

**References**	**Country**	**Methods**	**Participants/ Sample**	**Prevalence**	**Impact on Psychological Well-Being**
Lee et al. (52)	Western Pennsylvania and New Mexico	Phone survey	*N* = 49 participants, mean age: 56 years; gender: male 53%.	• (1) 27% of the participants had clinical levels of depressive symptoms, but only 12% had anxiety meeting clinical criteria. • (2) About 33% of participants reported poor sleep quality over the last month. Perceived stress was high in about 30% of participants, and 85% felt overwhelmed by difficulties with COVID-19, although 41% felt that things were pretty/very often going their way.	• (1) Anxiety; • (2) Depressive symptoms.
Sousa et al. (91)	Portugal	• Mixed method: (1) quantitative method from medical records, • (2) qualitative method semi-structured interviews	*N* = 20, mean age: 66.9 (±11.9); gender: male 55%.	• (1) Impact on family relationships (70%); fear of being infected due to high-risk condition (70%); increased emotional distress (55%); fear of getting infected in the dialysis unit (55%); difficulty adjusting to the contingency plan at the dialysis unit (55%); altered self-esteem and autonomy (40%). • (2) Impacts on disease and treatment-related health behaviors (25–55%), decreased physical activity (55%); management of dietary recommendations (35%); management of fluid restrictions (25%); need for nephrologist consultation (25%). • (3) Positive impacts (40%), personal growth (40%); increased social support (30%). • (4) Coping strategies (35-80%). • (5) Adherence to the protection measures at home (35%); engage in indoor and outdoor leisure activities (80%); seeking social support for instrumental use (65%); adherence to the protection measures at the dialysis clinic (55%); using social media and/or telephone to communicate (50%); religious coping (45%); seeking social support for emotional use (40%); avoidance(40%).	• (1) Emotional stress include anxiety; • Social distrust; • (3) Somatization.
Yang et al. (93)	China	(1) Survey	*N*= 273, mean age: 59.9 (+/− 14.4); gender: male 41.4%.	(1) Nonspecific psychiatric morbidity 45.8% by using General Health Questionniare-28 (GHQ-28) (2) Clinical concern (19.4%) by using Impact of Events Scale–Revised (IES-R) (3) Kidney Disease Quality of Life (KDQOL) and KDQOL-36 Short Form (SF) were significantly improved when compared with the intiital study (*p* = 0.006 and p = 0.031, respectively) (4) General Health Questionnaire-28 (GHQ-28) and Impact of Events Scale–Revised (IES-R) did not have significant change, but there were improvement in somatic symptoms (*p*= 0.006); anxiety and insomnia (*p*= 0.005); and intrusion (*p*=0.049)	(1) Somatic symptoms (2) Anxiety (3) Insomnia (4) Duration of hemodiaglysis may affect mental health, QoL, or health status.
Barutcu Atas et al. (49)	Turkey	(1) Survey	*N* = 106, mean age: 44.2 (±13.3), gender: male 61.3%.	• (1) High-perceived stress (49, 46.2%), • (2) Poor sleep quality (51, 48.1%) • (3) Insomina (40, 37.7%) • (4) Anxiety (25, 23.6%) • (5) Depression (47, 44.3%) • (6) Regression analyses revealed that high-perceived stress is an independent predictor of anxiety and depression.	• (1) Stress, • (2) Sleeping quality, • (3) Anxiety, • (4) Depression.

**Figure 1:**
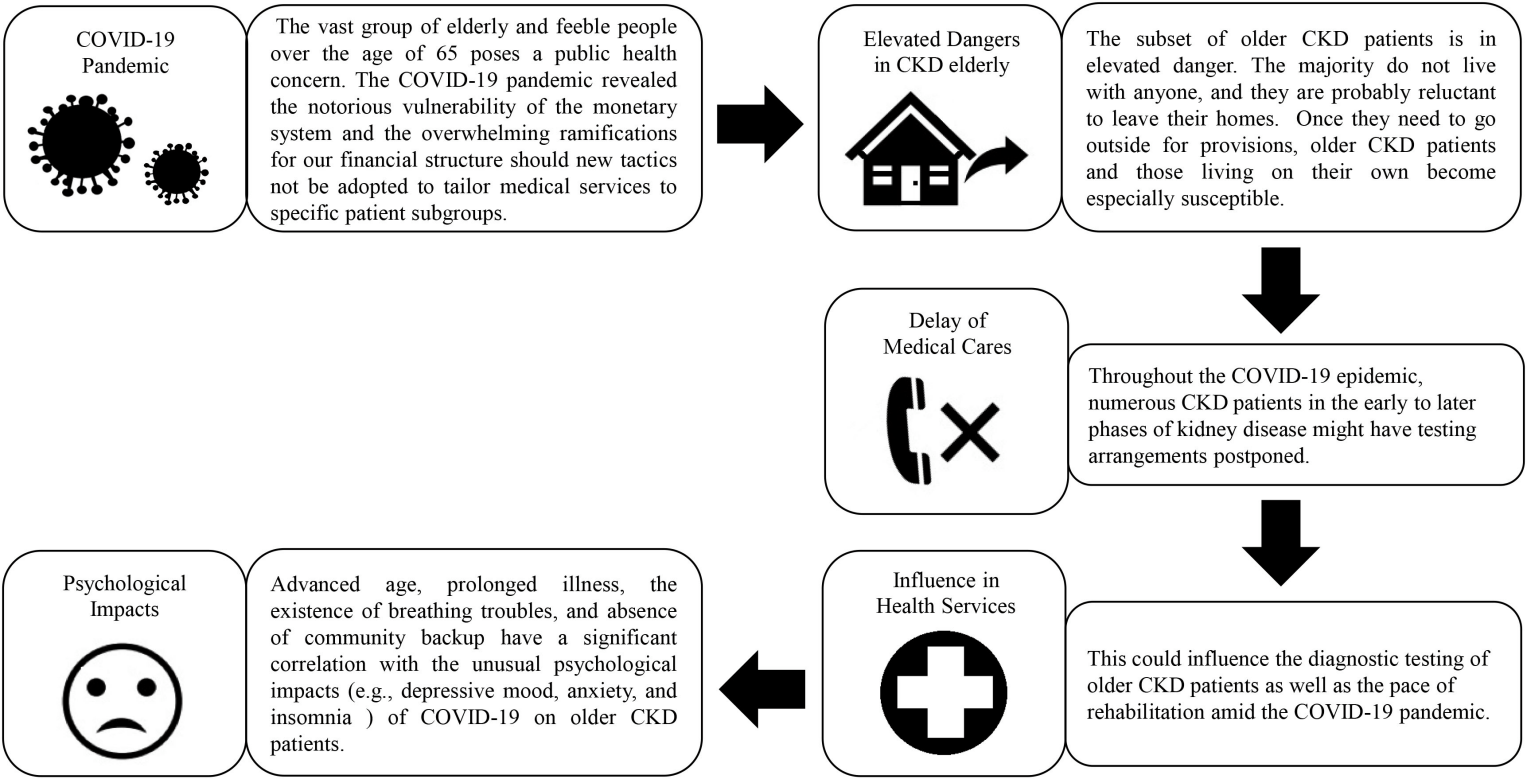
During COVID-19, the phenomenon has a detrimental psychological impact on CKD patients. Source: Coppolino et al. (64) and Varshney et al. (48).

In a multivariable study, Varshney et al. (48) found that advanced age, prolonged illness, the existence of breathing troubles, and absence of community backup have a significant correlation with unusual mental effects of COVID-19 on individuals with chronic disease. Once they need to go outside for provisions, elderly CKD patients and those living on their own become especially susceptible. Throughout the COVID-19 epidemic, numerous CKD patients in the early to later phases of kidney disease might have testing arrangements postponed. Failure to detect significant development of CKD has profound implications for both the patient and the community (Figure 1) (64, 91).

Healthcare practitioners may provide guidelines for mental stress and adjustment (such as planning exercises and timetabling) (92), connect patients with psychosocial health departments, and encourage patients to pursue adequate mental health assistance as needed (34). Nadler et al. (35) noted that because the caregivers typically lessen their children's discomfort, transparent talks should be encouraged to discuss children's reactions and issues. As far as health care providers themselves are concerned, the innovative concept of SARS-CoV-2, preliminary screening, minimal treatment options, insufficient PPE and other medical resources, prolonged unresolved pressures, and other related risks are sources of stress and could potentially overwhelm systems (36, 37). SARS-CoV-2 is spread from humans to humans through direct contact with an infected person *via* nasal spills or touching contaminated substances. Maturity and chronic illness have been identified as possible causes of severe disease and death (38). Ghinai et al. (39) confirmed that SARS-CoV-2, which resulted in the condition currently known as COVID-19, had been disseminated across China and 26 more countries as of 18 February 2020. Advanced age, being female, extended illness, breathing symptoms, and lack of social support were essentially related to the peculiar mental impact of COVID-19 on patients with renal impairment. Patients aged 34 or older were more likely to suffer from psychiatric disorders due to the recent outbreak (40, 41). This result invalidates an Indian study where young adults encountered more significant psychological problems due to COVID-19. This discrepancy may be attributed to the incorrect assumption that COVID-19 is not as accurate in younger individuals (42, 43).

Self-care offered by providers, like mental healthcare providers, requires training on disease and risks (44), tracking someone's pressure reaction, as well as seeking adequate assistance with personal and occupational responsibilities and issues, such as professional mental health (22, 45, 46). Healthcare systems must handle the burden of subcontractors and comprehensive operations by evaluating reactions and implementation, modifying projects and plans, adjusting expectations, and designing tools to deliver psychosocial assistance based on the circumstances (47).

## Discussion

The health care system must offer coaching and instruction on psychosocial problems to healthcare service administrators, emergency personnel, and health care providers. Mental health and emergency response systems must work together to identify, establish and allocate evidence-based resources such as disaster-related mental health, psychological well-being crisis and referral, special patient needs, and alarm and distress treatment. Risk consultation initiatives should resolve the difficulty of emerging problems, such as legislation, vaccination affordability and sufficiency, and the need for evidence-based arrangements related to disease outbreaks, and tackle various psychosocial considerations. Psychological well-being practitioners may strengthen perceptions that can be expressed through supporting the experts. The COVID-19 episode has a devastating impact on personal and collective welfare and care work. Despite health concerns, ultimately, medical treatment practitioners have a significant role in tracking psychosocial needs and delivering psychosocial assistance to their patients, providers of therapeutic services, and social initiatives that should be integrated into overall pandemic healthcare.

COVID-19 has contributed to increased recognized risk factors for mental health problems. In addition to weirdness and insecurity, quarantine and physical isolation can lead to significant alienation, lack of income, delays, limited access to core domains, increased exposure to alcohol and internet betting, and decreased family and community assistance, especially in more vulnerable people (21). The COVID-19 outbreak also provides a significant barrier to involvement in preliminary testing for older adults with tumors who are currently under-served in oncology and other clinical tests. Testing or possible admission to such therapeutic initial testing has been completed or based on several assessment projects worldwide.

## Conclusion

COVID-19 is an evolving and rapidly growing disease that warrants personalized attention and assessment depending on the incidence of the infection. When humanity is dealing with the outbreak and working to find ways to effectively distribute cancer treatment to more mature patients, it is necessary to intervene to protect the vulnerable and counter the prolonged detrimental consequences in this age group. Since this is unlikely the last outbreak in human history, it is crucial to embrace this opportunity to discover facts and formulate strategies for any possible scenarios. It should also be understood that previous studies could contribute to a range of uses depending on the stage of the outbreak. Overall, it is especially critical that older individuals practice social distancing. Nevertheless, the scientific evidence of the experiences of older adults has been minimal so far. To understand the impact of the outbreak on more mature people and to develop viable arrangements, it is vital to examine how older individuals respond to quarantine measures and identify the difficulties and frustrations faced by older people and patients with CKD.

## Author Contributions

AC and PT carried out the outline of this manuscript. AC wrote the manuscript with support from JH and JL. JH and JL gave valuable comments and suggestion. PT helped to supervise the whole manuscript with his professional advances in Chronic Kidney Disease. AC and PT linked up the situation of nowadays older CKD patients during COVID-19 and their risk factors of psychological well-being. All authors contributed to the article and approved the submitted version.

## Conflict of Interest

The authors declare that the research was conducted in the absence of any commercial or financial relationships that could be construed as a potential conflict of interest.
